# Enterochromaffin Cells: Sentinels to Gut Microbiota in Hyperalgesia?

**DOI:** 10.3389/fcimb.2021.760076

**Published:** 2021-10-14

**Authors:** Xiaolin Xu, Rongmin Chen, Gaofeng Zhan, Danning Wang, Xi Tan, Hui Xu

**Affiliations:** ^1^ Department of Anesthesiology, Tongji Hospital, Tongji Medical College, Huazhong University of Science and Technology, Wuhan, China; ^2^ Department of Anesthesiology, Huashan Hospital, Fudan University, Shanghai, China

**Keywords:** enterochromaffin cells, gut microbiota, hyperalgesia, chronic pain, microbiota-gut-brain axis

## Abstract

In recent years, increasing studies have been conducted on the mechanism of gut microbiota in neuropsychiatric diseases and non-neuropsychiatric diseases. The academic community has also recognized the existence of the microbiota-gut-brain axis. Chronic pain has always been an urgent difficulty for human beings, which often causes anxiety, depression, and other mental symptoms, seriously affecting people’s quality of life. Hyperalgesia is one of the main adverse reactions of chronic pain. The mechanism of gut microbiota in hyperalgesia has been extensively studied, providing a new target for pain treatment. Enterochromaffin cells, as the chief sentinel for sensing gut microbiota and its metabolites, can play an important role in the interaction between the gut microbiota and hyperalgesia through paracrine or neural pathways. Therefore, this systematic review describes the role of gut microbiota in the pathological mechanism of hyperalgesia, learns about the role of enterochromaffin cell receptors and secretions in hyperalgesia, and provides a new strategy for pain treatment by targeting enterochromaffin cells through restoring disturbed gut microbiota or supplementing probiotics.

## Introduction

Hyperalgesia refers to a decreased pain threshold and an increased response to harmful stimuli, which seriously affects patients’ health and quality of life (Nugraha et al., 2019). The central mechanisms of hyperalgesia mainly include glutamate/NMDA (N-methyl-D-aspartic acid) receptor-mediated sensitization, de-inhibition of inhibitory interneurons, and activation of microglia (Basbaum et al., 2009). Peripheral mechanisms include tissue damage and persistent inflammation (Treede et al., 1992). At present, many studies have proved that gut microbiota is involved in the peripheral regulation mechanism of chronic pain, and it is not only confined to visceral pain but also hyperalgesia induced by neuropathic and metabolic diseases (Pellegrini et al., 2018; Yang et al., 2019).

Diverse and stable gut microbiota is vital to the health of the host. The gut microbiota begins to establish soon after the host is born and is continuously affected by external factors, such as antibiotics, diet, stress, etc. The gut microbiota is a dynamic ecosystem that maintains a bidirectional connection with the host through the microbe-gut-organ axis and actively responds to various physiological and pathological conditions. Among them, studies on the mechanism of the microbe-gut-brain axis in neuropsychiatric diseases, including chronic pain and cognition, have attracted the most attention (Pellegrini et al., 2018; Zhan et al., 2018). Gut microbiota can affect the occurrence of visceral pain, inflammatory pain, neuropathic pain, headache, and opioid tolerance through peripheral and central mechanisms (Guo et al., 2019). Their regulatory role in chronic pain has opened up a new idea for pain treatment by restoring healthy gut microbiota (Xu et al., 2021).

The intestinal epithelium is composed of epithelial absorption cells, goblet cells, paneth cells, and enteroendocrine cells. Enterochromaffin cells (ECs) are the most abundant subtype of intestinal enteroendocrine cells in the colon, which can be directly contacted by gut microbiota on the side of the intestinal lumen and interact with the afferent and efferent nerve endings located in the lamina propria by synaptic connection. Although its accounts for less than 1% of intestinal epithelial cells, it can produce and release 90% of the serotonin in the body, which is essential for intestinal motility, platelet function, immune response, and bone development (Karsenty and Yadav, 2011; Matthes and Bader, 2018). In addition, ECs are electroexcitatory, similar to other primary sensory cells, expressing functional voltage-gated Na^+^ and Ca^2+^ channels (Bellono et al., 2017). These important characteristics enable ECs to act as bidirectional information transmitters among the intestinal lumen, intestinal epithelial cells, and specific primary afferent nerve fibers. In brief, ECs are considered the fundamental cells of microbiota-gut-brain interaction in cognition, depression, and chronic pain (Rhee et al., 2009).

## Gut Microbiota and Hyperalgesia

Gut microbiota has been recognized as one of the key pain regulators, which can directly or indirectly mediate pain tolerance or sensitization through immune, metabolic, endocrine, and neural signaling pathways. Numerous clinical or pre-clinical studies on chronic pain-related diseases have reported changes in gut microbiota (Braundmeier-Fleming et al., 2016; Clos-Garcia et al., 2019). For example, the diversity of the gut microbiota in patients with fibromyalgia was reduced, and the abundance of *Bifidobacterium* and *Eubacterium* genera was significantly reduced (Clos-Garcia et al., 2019). *Eggerthella sinensis, Colinsella aerofaciens, Faecalibacterium prasunitzii, Odoribacter splanchnicus*, and *Lactonifactor longoviformis* decreased in patients with bladder pain syndrome (Braundmeier-Fleming et al., 2016). In addition, a large cohort study proved that the abundance of *Streptococcus* species was linked to the increase in knee joint pain (Boer et al., 2019). PubMed was searched, and we selected relevant studies before August 1st, 2021. The search keyword string included “hyperalgesia OR chronic pain” AND “gut microbiota OR intestinal flora.” To this end, 15 related studies were enrolled. The details of each study are presented in **Table 1**.

**Table 1:** Summary of studies investigating links between hyperalgesia and the gut microbiota.

Pathology type	Study Design (subjects, intervention)	Bacterie/Bacterial metabolites (algesia-resilience & healthy)	Bacterie/Bacterial metabolites (algesia-sensitivity & pain)	Reference
**Human Studies**				
Back Pain	36 overweight or obese patients	*Dialister*, *Lactobacillus*↑	genera *Adlercreutzia*, *Roseburia, Uncl*. *Christensenellaceae*↑	(Dekker Nitert et al., 2020)
CWP	female 113 CWP patients and 1623 controls	/	family *Lachnospiraceae*, family *Ruminococcaceae*↓; family *Lachnospiraceae*↑	(Freidin et al., 2021)
Fibromyalgia	105 fibromyalgia patients and 54 healthy individuals	/	family *Bifidobacterium*, *Eubacterium*, *Lachnospiraceae*↓, phylum *Clostridium*, *Firmicutes*↓; *Dorea*, *Roseburia*, *Papillibacter*, *Subdoligranulum*↑; PAF-16↓, l-glutamine, l-threonine/DL-homoserine, l-arginine, ADMA, l-glutamate, Nϵ-methyl-l-lysine, Ornithine↑	(Clos-Garcia et al., 2019)
IBS	*253 IBS patients and 186 controls*	*Ruminococcaceae UCG-005*, *Holdemanella*, *Coprococcus 2*, *Eubacterium coprostanoligenes group*↑	*Lachnoclostridium*, *Dorea*, *Erysipelatoclostridium*, *Prevotella 9*, *Clostridium sensu stricto 1*↑	(Liu et al., 2021b)
IBS	*15 IBS patients and 15 healthy controls*	*/*	*Lachnospira*, *Clostridium*↑; L-methionine and homocysteine↑	(Zhu et al., 2019)
IBS	*22 IBS children and 22 healthy children*	genus *Eubacterium*, species *Bacteroides vulgatus*↑	phylum *Proteobacteria*, class *Gammaproteobacteria*, genera *Dorea*, genera *Haemophilus*, Species *H. parainfluenzae* and a novel *Ruminococcus*-like organism↑	(Saulnier et al., 2011)
IC	*female 17 patients and 17 controls*	/	*Eggerthella sinensis*, *Colinsella aerofaciens*, *Faecalibacterium prasunitzii*, *Odoribacter splanchnicus* and *Lactonifactor longoviformis*↓	(Braundmeier-Fleming et al., 2016)
Joint pain	*124 patients and 817 controls*	/	*Streptococcus*↓	(Boer et al., 2019)
**Animal Studies**				
CCI	*5- to 6-week-old male SD rats*	phylum *Proteobacteria*, *Bacteroidetes*, *Cyanobacteria*, *Actinobacteria*, *Firmicutes*↑	phylum *Firmicutes*, *Actinobacteria*, *Proteobacteria*↑; genus *Lactobacillus*, *Helicobacter*, *Blautia*, *Christensenella*, *Phascolarctobacterium*, *Streptococcus*, *Rothia*↑, *Escherichia*, *Corynebacterium*, *Ignatzschineria*, *AF12*, *Butyricimonas*↓	(Chen et al., 2021a)
CINP	8 weeks female C57BL/6J mice (oxaliplatin, hydrogen-rich water)	phylum *Firmicutes*, *Tenericutes*↑, *Bacteroidetes*↓; family *Lachnospiraceae*, *Lactobacillaceae*, *Ruminococcaceae*↑; genus *Faecalibacterium*, *Lactobacillus*, *Roseburia*↑	/	(Lian et al., 2021)
Neuropathic pain	*8-12 weeks female C57BL/6 mice (SNI)*	*Oscillospira*, *Erysipelotrichaceae_unclassified*, *Adlercreutzia* and *Turicibacter*↑	*Staphylococcus*, *Ruminococcaceae_uncultured* and *Turicibacter*↑	(Brandon-Mong et al., 2020)
Tactile allodynia	*3 weeks male C57BL/6 mice (low vitamin D concentration)*	/	*Firmicutes*↑, *Verrucomicrobia*, *Bacteroidetes*↓	(Guida et al., 2020)
TMDs	*8 weeks male C57BL/6 mice (CFA)*	/	*Bacteroidetes* and *Lachnospiraceae*↓	(Ma et al., 2020)
Visceral hyperalgesia	*male Wistar rats (WAS/RS)*	*Lactobacillus*↑; *Clostridiaceae*, *Erysipelotrichaceae* and *Peptostreptococcaceae*↓ (after rifaximin treatment)	*/*	(Xu et al., 2014)
Visceral hyperalgesia	*male Wistar rats (WAS/RS)*	*Lactobacillaceae*↑; *segmented filamentous bacteria*↓ (after rifaximin treatment)	*/*	(Gao et al., 2014)

↑, indicates increase; ↓, indicates decrease.

AR, algesia-resilience; AS, algesia-sensitivity; CCI, chronic constriction injury; CFA, complete Freund’s adjuvant; CINP, chemotherapy-induced neuropathic pain; CPP, chronic postoperative pain; CWP, chronic widespread musculoskeletal pain; IBS, irritable bowel syndrome; IC, interstitial cystitis/bladder pain syndrome; SNI, spared nerve injury; TMDs, temporomandibular disorders; RS, repeat restraint stress; WAS, chronic water avoidance stress.

In studies related to gut microbiota, the use of antibiotics to construct pseudo-sterile mouse models is a conventional intervention (Yang et al., 2019; Zhang et al., 2019). The common measuring method of behavioral pain response included: mechanical sensitivity by von Frey method, cold sensitivity by cold plantar assay, heat sensitivity by Hargreaves test, or tail-flick test. The imbalance of the gut microbiota caused by antibiotics also impacts the occurrence of pain. However, the effects of antibiotic-induced changes in the gut microbiota on pain have been inconsistent in numerous studies (Shen et al., 2017; Yang et al., 2019). It has been suggested that the lack of gut microbiota may be a protective mechanism in terms of hyperalgesia for the host. Compared with wild mice, inflammatory stimuli, such as carrageenan, lipopolysaccharide (LPS), tumor necrosis factor (TNF)- alpha, interleukin (IL)-1beta, and chemokine (C-X-C motif) ligand 1 (CXCL1), cause relatively less noxious reactions in germ-free (GF) mice, while recolonization of the gut microbiota or administration of IL-10 antagonists can reverse this phenomenon in GF mice (Amaral et al., 2008). Similarly, mechanical hyperalgesia in neuropathic pain induced by chemotherapy drugs (paclitaxel, oxaliplatin) and chronic compressive nerve injury pain was reduced in GF mice or antibiotic-treated mice, while recovery of the gut microbiota can disrupt this protective effect and promote chemotherapy-induced mechanical hyperalgesia (Shen et al., 2017; Ramakrishna et al., 2019; Ding et al., 2021). Conversely, some studies have concluded that the interference of antibiotic administration leads to the imbalance of the gut microbiota in mice, which can induce mechanical tenderness and spontaneous pain, accompanied by anxiety, depression-like behaviors, and spatial memory impairment (Aguilera et al., 2015; Yang et al., 2019). Transplanting the gut microbiota from spared nerve injury model rats susceptible to anhedonia into antibiotic treatment rats further aggravated chronic pain and depression-like phenotypes (Yang et al., 2019). In addition, in mice with dysbacteriosis, the cannabinoid receptor (CB) 2 level was upregulated, while the CB1 and μ opioid receptors expressions were down-regulated (Aguilera et al., 2013; Aguilera et al., 2015), and large amounts of IL-10 could be produced (Souza et al., 2004), affecting the pain response. Although there are different opinions about the effect of gut microbiota imbalance on chronic pain, which may involve differences in the antibiotic compatibility, detection time, dosage, and even animal models, these experimental results are sufficient to prove that the symbiotic gut microbiota affects the production of hyperalgesia in the host.

Access to effective bacteria or prebiotics is one of the crucial research goals in treating various systemic diseases, and chronic pain is no exception. *Lactobacillus paracasei* and *Bifidobacterium infantis 35624* can normalize pain threshold (Verdú et al., 2006; McKernan et al., 2010). Taking *Lactobacillus acidophilus NCFM* strain can induce the expression of μ opioid receptor and CB2 in intestinal epithelial cells, mediating the analgesic effects similar to morphine (Rousseaux et al., 2007). Intestinal microbial exopolysaccharides (EPSs) have been shown to play an active role in antioxidant, blood pressure and blood glucose regulation, apoptosis, and autophagy of cancer cell lines (Maeda et al., 2004; Di et al., 2017; Tang et al., 2017). Intraperitoneal injection of probiotic strain *Lactobacillus paraplantarum BGCG11* (EPS CG11) producing high molecular weight EPSs can significantly reduce the mechanical hyperalgesia of inflammatory pain in Wistar rats by decreasing the expression of pro-inflammatory factors IL-1β and inducible nitric oxide synthase (iNOS) (Dinić et al., 2018) (**Table 2**)

**Table 2:** Summary of probiotics associated with the underlying mechanisms of pain.

Probiotics	Pathology type	Study Design (subjects, intervention)	Function	Potential mechanisms related to pain	Reference
SLAB51	CIPN	male CD1 mice (PTX)	prevented the mechanical and cold hypersensitivity	CB-1, µ, κ receptors, PPARγ protein↑, p-Stat3, p-Jak2, p-FAK, acetylated α-tubulin in the spinal cord↓; COX-2, Inos, TNF-α, IL-1β, IL-6 in the serum↓, IENFs in the paw↑	(Cuozzo et al., 2021)	
*Lactobacillus reuteri*	Colon obstruction	8-10 weeks male Sprague-Dawley rats	decreased sensory neuron hyperexcitability and referred hyperalgesia in colon obstruction	Restored μ, δ, κ receptors (MOR-1, DOR-1, KOR-1, respectively)	(Hegde et al., 2020)	
*Lactobacillus reuteri* DSM 17938	Functional abdominal pain	children	reduced the intensity of pain and increased the pain-free days	None	(Romano et al., 2014; Weizman et al., 2016; Jadrešin et al., 2017; Jadrešin et al., 2020)	
*Lactobacillus acidophilus* NCFM	Functional abdominal pain	20 caucasian women with mild to moderate abdominal pain	reduced visceral sensitivity	modulates mu-opioid receptor expression and activity	(Ringel-Kulka et al., 2014)	
*Bacillus coagulans* GBI-30, 6086	IBS	44 IBS patients (probiotics: 22, placebo: 22)	relieved of abdominal pain and bloating	None	(Hun, 2009)	
*Lactobacillus plantarum* PS128	IBS	8 weeks male Sprague–Dawley rats	reduced visceral hypersensitivity	substance P, CGRP, BDNF, NGF in the dorsal root ganglion↑ but in the spinal cord↓; corticosterone in serum and mineralocorticoid receptors in the amygdala↓	(Liu et al., 2020)	
*Saccharomyces cerevisiae* I-3856	IBS	379 IBS patients	does not improve intestinal pain and discomfort	None	(Spiller et al., 2016)	
*Bifidobacterium bifidum* MIMBb75	IBS	122 IBS patients (placebo: 62, MIMBb75: 60)	improved pain/discomfort, distension/bloating, urgency and digestive disorder	None	(Guglielmetti et al., 2011)	
*Bifidobacterium longum*, *B. bifidum*, *B. lactis*, *Lactobacillus acidophilus*, *L. rhamnosus*, *Streptococcus thermophilus*	IBS	49 IBS patients (probiotics: 25, placebo: 24)	improvement in abdominal pain/discomfort and bloating	*B. lactis*, *L. rhamnosus*, and *S. thermophilus*↑ in the probiotics group after 4 weeks and that *B. lactis*↑ in the placebo group.	(Yoon et al., 2014)	
VSL#3 (a mixture of 8 probiotic bacteria strains)	IBS	male Wistar rats (neonatal maternal separation)	reversed both allodynia and hyperalgesia in neonatal maternal separation Rats	VSL#3 counter-regulated genes (CCL2, NOS3, IL10 and TNFRSF1B) involved in the inflammatory cascade and genes (TLRs, NFκB and MAPKs) that encode for factors that regulate the innate and adaptive immune response, thus inhibiting inflammatory and nociceptive processes	(Distrutti et al., 2013)	
*Lactobacillus rhamnosus*	Osteoarthritis	6 weeks male Wistar rats (MIA)	decreased pain severity and cartilage destruction	decreased Intestinal damage and inflammation	(Jhun et al., 2021)	
Isomalto-oligosaccharides	VHS	male Wistar rats (WAS)	increased pain threshold	repaired damage of intestinal epithelial ultrastructure	(Wang et al., 2017b)	
*Lactobacillus plantarum*	VHS	male Sprague-Dawley rats (colorectal distensio)	D-alanine depletion of lipoteichoic acid in *Lactobacillus plantarum* inhibited visceral pain perception	decreased the activation-induced release of TNF and IFN-gamma from mesenteric T cells and the IL-10 concentration in colonic tissue, while increasing the activation-induced secretion of IL-10 in splenocytes and mesenteric lymphocytes and the baseline IL-10 release ofsplenocytes.	(Duncker et al., 2008)	
*Lactobacillus paracasei* (NCC2461)	VHS	female NIH Swiss mice (bacitracin, neomycin, primaricin)	attenuates antibiotic induced visceral hypersensitivity	normalized visceral sensitivity and substance P immunolabelling	(Verdú et al., 2006)	

↑, indicates increase; ↓, indicates decrease.

CIPN, chemotherapy-induced peripheral neuropathy; COX-2, cyclooxygenase-2; FSH, follicle-stimulating hormone; IENFs, intra-epidermal fiber; iNOS, inducible nitric oxide synthase; MIA, monosodium iodoacetate; NLB, neonatal limited bedding; PPARγ, peroxisome proliferator-activated receptor gamma; PTX, paclitaxel; VHS, visceral hypersensitivity; WAS, water avoidance stress.

In addition, the imbalance of gut microbiota can lead to structural and metabolic changes in the chronic pain-related brain areas. Anterior cingulate cortex (ACC) volume was decreased, periaqueductal grey volume was increased, and single nerve cells in the ACC also appeared noticeable dendrite changes in pseudo-sterile mice with visceral sensitization (Luczynski et al., 2017). The number of C-Fos immunoreactive neurons in the prefrontal cortex and hippocampus was decreased, while in ACC and insular cortex was increased in mice with dysbacteriosis (Wang et al., 2021a). The amygdala also plays a role in mood disorders, nerve regeneration diseases, and chronic pain. In particular, humans have labeled the laterocapsular division of the central nucleus as “pain-sensitive amygdala.” The gut microbiota can affect the morphology, activity, functional connection, and gene expression of the amygdala through the vagus nerve [including the enteric nervous system (ENS)], spinal cord transmission (especially visceral pain), regulation of tryptophan metabolism, and immune regulation (Cowan et al., 2018). The activation of microglia is also associated with the development of chronic pain. The microglia in the brain mainly initiate neuronal apoptosis, clearing dead cells and pruning synapses. However, microglia in antibiotic treatment or GF mice show immature and malformed phenotypes with significantly longer processes and increased numbers of segments, branching, and terminal points. In addition, mice deficient for the short-chain fatty acids (SCFAs) receptor, free fatty acid receptor (FFAR) 2, also showed an immature state of microglia in pseudo-sterile mice, indicating that the presence of gut microbiota is critical for the development, homeostasis, and functional status of microglia in the central nervous system (Erny et al., 2015). A considerable part of the metabolites in mammalian blood is derived from gut microbiota, and changes in the gut microbiota will also affect brain metabolites (Wikoff et al., 2009). Furthermore, GF mice were found to have lower tryptophan (a precursor of 5-HT), tyrosine (a precursor of dopamine and norepinephrine), and glutamine in the brain than mice rich in gut microbiota, in addition to lower energy production and consumption through glycolysis and the tricarboxylic acid cycle (Matsumoto et al., 2013). In GF mice, the blood-brain barrier (BBB) permeability was increased, and the expressions of tight junction proteins such as occludin and claudin-5 in the frontal cortex, striatum, hippocampus were decreased (Braniste et al., 2014). While *Clostridium tyrobutyricum* or *Bacteroides thetaiotaomicron* treatment can increase tight junction protein expression and restore BBB permeability in GF mice, which indicated that the gut microbiota was contributed to maintaining the integrity of the BBB, and the destruction of BBB caused by gut microbiota imbalance provided a structural basis for harmful intestinal substances to affect the host brain function (Braniste et al., 2014). The above suggested that the gut microbiota is inextricably linked to the host’s hyperalgesia (**Table 3**).

**Table 3:** The studies associated with changes in brain regions and gut microbiota.

Study Design (subjects, intervention)	Bacteries	Changes in brain region (intestinal dysbacteriosis)	Reference
GF and SPF NMRI mice	/	noradrenaline, dopamine, and 5-HT turnover↑ in the striatum; NGFI-A mRNA↓ in the orbital frontal cortex, striatum, hippocampus (CA1,CA3 region, dentate gyrus), amygdala; BDNF mRNA↓ in the hippocampus, amygdala, cingulate cortex in GF mice compared with SPF mice. Synaptophysin and PSD-95 in the striatum↓ in SPF and CON mice compared with GF mice.	(Diaz Heijtz et al., 2011)
5 weeks male C57BL/6 mice (bacitracin, neomycin, natamycin, meropenem, vancomycin)	*Firmicutes*, *Bacteroidetes*↓, *Proteobacteria*↑	tight-junction proteins↓ of the brain blood vessels and BBB permeability↑ in antibiotic treated mice	(Sun et al., 2021)
male Wistar rats (T2DM; *Lactobacillus plantarum*, inulin, or synbiotic)	dominant populations were *lactobacillus*	*Lactobacillus plantarum* led to a significant decrease in TLR-2 as well as GDNF and GFAP only in the amygdala	(Hosseinifard et al., 2019)
*Healthy women (FMPP: 12, controls: 11, no intervention: 13)*	/	FMPP reduces the reactivity of a widely distributed network of brain regions (primary interoceptive and somatosensory regions, and a cluster in the midbrain region centered on the periaqueductal gray, the prefrontal cortex, precuneus, basal ganglia, and the parahippocampal gyrus) to an emotional attention task.	(Tillisch et al., 2013)
Swiss Webster (GF and CON mice); Adult male Sprague Dawley rats (ampicillin, vancomycin, ciprofloxacin HCL, imipenem, metronidazole)	/	103 miRNAs (61 downregulated, 42 upregulated) changed in the amygdala, and 31 miRNAs (21 downregulated, 10 upregulated) altered in the PFC in GF animals	(Hoban et al., 2017)
BALB/c mice (SPF and GF mice)	/	l-aspartic acid in striatum, cerebral cortex and hippocampus, and l-arginine, l-alanine and l-valine in striatum↑ in SPF mice than in GF mice	(Kawase et al., 2017)
12 weeks C57BL/6J mice (SPF and GF mice)	/	microbiota dependent-hypomyelination of several gray matter structures (neocortex, HIP, brainstem) and major white matter tracts (the corpus callosum, anterior commissure, internal capsule) specifically in GF mice using MPF imaging.	(Lu et al., 2018)
*Swiss Webster (GF and CC mice)*	/	the volumes of ACC↓and periaqueductal grey↑, dendritic changes in the ACC were evident in GFmice.	(Luczynski et al., 2017)
*6 weeks male C57BL/6J mice (ampicillin, streptomycin, clindamycin)*	*Bifidobacterium*, *Escherichia Coli*, *Lactobacillus*↓	Fos immunoreactive (ir) neurons in mPFC and HIP↓, in ACC and IC↑ in antibiotic treated mice	(Wang et al., 2021b)
GF mice (E. coli JM83, complex microbiota, or no microbiota)	/	displayed disorganization of gene co-expression networks in HIP, amygdala, mPFC in E. coli JM83 treated mice	(Philip et al., 2021)

↑, indicates increase; ↓, indicates decrease.

ACC, anterior cingulate cortex; BBB, blood-brain barrier; CC, conventionally colonized; FMPP, fermented milk product with probiotic, containing Bifidobacterium animalis subsp Lactis, Streptococcus thermophiles, Lactobacillus bulgaricus, and Lactococcus lactis subsp Lactis; GDNF, glial cell-derived neurotrophic factor; GFAP, glial fibrillary acidic protein; GF, germ-free; HIP, hippocampus; IC, insular cortex; PFC, prefrontal cortex; SPF, specific pathogen -free; T2DM, type 2 diabetes mellitus.

## Gut Microbiota Medium or Metabolites and Hyperalgesia

The role of mediators or metabolites derived from gut microbiota in hyperalgesia is well known. Gut microbiota can release nervous system factors involved in the regulation of gut brain-axis communication, such as 5-HT released by genera *Candida*, *Streptococcus*, *Escherichia* and *Enterococcus*, dopamine or norepinephrine produced by genera *Escherichia*, *Bacillus* and *Saccharomyces*, acetylcholine generated by genus *Lactobacillus*, γ-aminobutyric acid produced by genera *Lactobacillus* and *Bifidobacterium* (Holzer and Farzi, 2014). Moreover, gut microbiota-derived mediators can activate Toll-like receptors (TLRs), GABA receptors, and transient receptor potentials (TRP), and so on, participating in the regulation of chronic pain (Santoni et al., 2021).

Pathogen-associated molecular patterns (PAMPs) derived from gut microbiota were significant contributors to peripheral sensitization under chronic pain conditions. PAMPs obtained from gut microbiota included LPS, flagellin, N-formyl peptides, lipoteichoic acid, peptidoglycan, β-glucan, etc. (Chiu, 2018). They can directly sensitize the primary neurons in the dorsal root ganglia or indirectly by activating immune cells to promote the release of cytokines and chemokines, mediating peripheral sensitization of pain. LPS can send signals to nociceptive dorsal root ganglion neurons in the colon of mice (Ochoa-Cortes et al., 2010). Bacterial flagella can be recognized by the host TLR5 to play a host defenses role (Hayashi et al., 2001) and block the sensitization of dorsal root ganglion A fiber sensory neurons, inhibiting the mechanical hyperalgesia caused by chemotherapy, nerve injury, and diabetic neuropathy (Xu et al., 2015). N-formyl peptide bound to the formyl peptide receptors on the host nociceptive dorsal root ganglia to induce mechanical hyperalgesia (Chiu et al., 2013).

In addition to PAMPs, gut microbiota metabolites are also involved in pain regulation. Microbiota-derived metabolites include SCFAs, bile acids, indole derivatives, vitamins, polyamines, and lipids (Hosseinkhani et al., 2021). Gut microbiota was considered the main producer of SCFAs, providing SCFAs to the host by decomposing fermented starch and dietary fiber. SCFAs included volatile fatty acids fermentation products such as isovaleric acid, isobutyric acid, and butyric acid. Several receptors of SCFAs have been identified as G-protein coupled receptor (GPR) 41/FFAR3, GPR43/FFAR2, GPR109A, and olfactory receptor (Olfr) 78, which mediate leukocyte recruitment, chemokine production, intestinal wall permeability, and BBB permeability changes (Braniste et al., 2014; Ohira et al., 2017). In chronic constriction injury, obesity-induced peripheral neuropathy pain, rheumatoid arthritis pain, changes in SCFAs and butyrate (a histone deacetylase inhibitor) for pain relief were shown. The mechanism involved alleviating the polarization of pro-inflammatory microglia in the spinal cord and hippocampus (Zhou et al., 2021) and increasing the serotonin metabolite 5-hydroxyindole-3-acetic acid, which in turn activated the aryl-hydrocarbon receptor and inhibited inflammation and pain in a Breg cell-dependent manner (Rosser et al., 2020), changing in the immune cell population of the peripheral nervous system (Bonomo et al., 2020).

## The Interaction of Hyperalgesia-Related Receptors in ECs With Intestinal Flora and Its Metabolites

ECs, as intestinal epithelial chemoreceptors, can detect gastrointestinal symbiotic bacteria, infectious microorganisms, food intake, endogenous regulatory substances, etc. It has been found that allyl isothiocyanate, isovalerate, isobutyrate, butyrate, catecholamines, and son on can specifically and continuously activate ECs, trigger Ca^2+^ transients, and participate in a variety of pathophysiological states (Bellono et al., 2017). From the perspective of chronic pain-related receptors of ECs, the possible hyperalgesia targets of ECs interacting with gut microbiota are discussed below.

### TRP Family

The TRP family is a kind of non-selective transmembrane cation channels superfamily, which can be divided into seven subfamilies according to different amino acid sequences: TRPC (canonical), TRPV (vanilloid), TRPM (melastatin), TRPP (polycystin), TRPML (mucolipin), TRPA (ankyrin) and TRPN (NOMPC like, or no mechanoreceptor potential C). They maintain the transmembrane transport of cell Na^+^, Ca^2+^ and Mg^2+^, and intracellular organelle homeostasis, participating in various pathophysiological processes such as neurodegenerative diseases and gastrointestinal peristalsis (Nilius and Owsianik, 2011; Blackshaw, 2014; Nilius and Szallasi, 2014). Among them, at least TRPV1-4, TRPM8 and TRPA1 are expressed in nociceptive sensory neurons, which transfer thermal, chemical, and mechanical stimulation signals, playing an important role in the occurrence and development of pathological pain perception (Dai, 2016). TRPV1 (Deng et al., 2021), TRPV5 and 6 (Hua et al., 2019), TRPM7 (Lv et al., 2020) and 8 (Wen et al., 2020), TRPA1 (Pagano et al., 2019), TRPP1 (Beer et al., 2019) have all been found to have some associations with the gut microbiota, but so far only the TRPA1 receptor has been studied in ECs (Nozawa et al., 2009).

TRPA1 is a chemoreceptor widely expressed in humans and animals, including dorsal root ganglia, bladder, gastrointestinal tract, skin, respiratory tract, blood vessels, etc. It helps to sense pain, temperature (<17°C), mechanical stimulation, and chemical stimulants. It has become a target for the development of analgesic and anti-inflammatory drugs. Its functional mutations were considered one of the pathogeneses of familial paroxysmal pain syndrome (Kremeyer et al., 2010). TRPA1 receptor is activated by allyl isothiocyanate, cinnamaldehyde, organic sulfur compounds in garlic and onions, and smoke bombs (Bautista et al., 2006). In the gastrointestinal tract, the TRPA1 receptor is mainly expressed in the visceral afferent nerves, which can sense exogenous dietary stimuli such as mustard and garlic and endogenous inflammatory factors such as prostaglandins and other lipid-derived metabolites (Logashina et al., 2019). As the main detector of luminal irritants on the intestinal mucosa, the TRPA1 receptor is necessary for the normal mechanical and chemical sensory functions of specific subsets of the vagus, viscera, and pelvic afferent nerve (Brierley et al., 2009). It has been demonstrated that TRPA1 is a molecular target of LPS, a toxic substance produced by bacterial lysis, that directly acts on nociceptive sensory neurons. This finding provides new insight into the mechanism of hyperalgesia during bacterial infection (Meseguer et al., 2014). IL-33 can be expressed and released by damaged tissues or necrotic barrier cells to participate in intestinal infections and type II immune responses. The IL-33/ST2 signaling pathway is an important signal to activate the dorsal horn ganglion and induce pain and itch (Han et al., 2013). IL-13/ST2 signaling pathway can mediate the rapid release of 5-HT from ECs dependent on the TRPA1 receptor and then cause the corresponding symptoms of 5-HT dysregulation (Chen et al., 2021b). In addition, cinnamaldehyde can also stimulate the QGP-1 cells of the human ECs model to release 5-HT in a dose-dependent manner by activating TRPA1 (Lieder et al., 2020).

### Piezo1/2

Since piezo1/2 was identified in mammalian cells in 2010 (Coste et al., 2010), the research focus of piezo1/2 has expanded from its structure to activation mechanism and its role in physiology and pathology (Kim et al., 2012; Guo et al., 2021). Piezo1/2 is a new type of mechanical ion channel that can be expressed in neurons, endothelial cells, red blood cells, etc. It acts as a multi-functional mechanical sensor in the bladder, colon, kidney, lung, and skin. Mechanical stimulus signals, such as tension and pulsation, are converted into electrochemical signals, which play an important role in blood vessel development, bone formation, and somatosensory conduction (Ranade et al., 2014a; Ranade et al., 2014b; Syeda et al., 2016; Jiang et al., 2021). Piezo1 is predominantly expressed in non-sensory tissues, while piezo2 is mainly expressed in sensory tissues. Some early studies focused on the role of piezo1/2 in the trigeminal nervous system (Fernández-Trillo et al., 2020; Dolgorukova et al., 2021). For example, IL-6 can cause trigeminal neuralgia by activating piezo2 (Liu et al., 2021a). In addition, activating piezo1 receptor on trigeminal nerve nociceptive fibers can trigger the release of calcitonin gene -related peptide, a key mediator of migraine (Mikhailov et al., 2019). The mechanisms of epac1-piezo2 axis in bone cancer pain, inflammatory pain, and mechanical allodynia of neuropathic pain have also been investigated (Eijkelkamp et al., 2013; Singhmar et al., 2016; Ni et al., 2021). Piezo1/2 also exists in intestinal epithelial cells, and the expression level in the colon is higher than that in the small intestine. Piezo2 in the colon is significantly negatively correlated with the visceral sensitivity of irritable bowel syndrome (IBS) (Bai et al., 2017). Selectively and high expression of piezo2 in human and mouse ECs can sense mechanical stimulation and induce the release of 5-HT (Wang et al., 2017a). More interestingly, piezo1 can induce ECs to produce 5-HT by sensing the single-stranded RNA (ssRNA) of the gut microbiota (rather than protein and DNA). ssRNA-stimulated Piezo1 induced a significant calcium response to release 5-HT in a MyD88/TRIF-independent and canonical Wnt signaling-independent manner, promoting intestinal motility and reducing bone mass, etc., independently of the stimulation of mechanical intestinal peristalsis (Sugisawa et al., 2020). Although the mechanism by which ssRNA of the gut microbiota activates piezo1/2 has still not been fully explained, the groundbreaking discovery of the interaction between the gut microbiota and piezo1/2 in ECs could be a theoretical cornerstone for treating hyperalgesia with gut microbiota.

### Olfactory Receptor (Olfr)

Although Olfrs are a type of GPR mainly responsible for the volatile odor signals transduction in olfactory neurons, they can be ectopically expressed in non-olfactory tissues, such as the heart, skin, lungs, and intestinal epithelium, etc., with both olfactory and non-olfactory functions (Braun et al., 2007; Chen et al., 2018). Olfr plays an important role in many physiological and pathological processes such as sensory perception, behavior and emotion regulation, immune system activity and inflammation regulation, tumor growth, and metastasis (Meijerink, 2021). Although there were few studies on its mechanism of action in hyperalgesia, the current research status suggests that it is closely related to hyperalgesia in certain diseases (Lee et al., 2019). The genome-wide association study of blood samples from breast cancer patients showed that the occurrence of pain after adjuvant radiotherapy was significantly associated with the activity of Olfr genes (OR52N1, OR4C12, OR4A47) (Lee et al., 2019). Whole-genome analysis of whole blood samples from patients with head and neck tumors showed that Olfr genes (OR13G1, OR6F1, OR14A2) were susceptibility genes for pretreatment pain (Reyes-Gibby et al., 2016). In addition, R-carvone-responsive Olfr OR1A1 has been used in a pre-clinical study to design cells controlled by peppermint aromatherapy to treat chronic pain (Wang et al., 2018). In recent years, Olfr78/OR51E2 and Olfr558/OR51E1, members of the olfactory GPR subfamily, have been identified as sensors for SCFAs and/or branched-chain fatty acids in the intestine. Olfr78, the SCFAs receptor on enteroendocrine cells, shows a specific affinity for acetic acid and propionic acid. It can promote the secretion of anorexigenic gut hormone peptide YY in mice intestinal enteroendocrine cells, regulate appetite, and maintain energy homeostasis (Nishida et al., 2021). Propionic acid can regulate blood pressure in mice by regulating renin release and vascular tension, proving a subtle relationship between gut microbial metabolites and Olfr78 on vascular smooth muscle cells in blood pressure control (Pluznick et al., 2013; Pluznick, 2014). In addition, acetic acid and propionic acid can also act on the Olfr OR51E2 (human-derived Olfr78) on airway smooth muscle cells to slow down the remodeling of cytoskeleton and the proliferation of airway smooth muscle cells, becoming specific receptors targeting the intestine-lung axis to treat asthma (Aisenberg et al., 2016). These suggest that certain specific connections between the gut microbiota with its metabolites and Olfrs play a role in the physiological and pathological processes. In addition, the intestinal odors can stimulate the release of 5-HT through the Olfrs localized at the apical side of ECs (Braun et al., 2007) to participate in the occurrence of gastrointestinal diseases such as IBS. Recently, it has been further discovered that isovaleric acid can act as a ligand for Olfr558, which can activate Gαolf/s-adenylyl cyclase signaling in ECs, induce 5-HT_3_ secretion involved in the occurrence of visceral sensitivity (Bellono et al., 2017). Therefore, the excavation of the mechanism of Olfrs on ECs will be more conducive to understanding the relationship between the gut microbiota and various pain-related diseases.

### α2Aadrenoreceptor

Research on the role of gut microbiota and its metabolites acting through adrenergic receptors is mainly manifested in lipid metabolism and cardiovascular aspects, such as promoting platelet thrombosis (Huynh, 2020). Changes in the gut microbiota will also affect the host catecholamine hormone levels. It was found that the level of free catecholamines in the intestine of pseudo-sterile mice was lower than that of specific pathogen-free mice, and most of the catecholamines in the intestines of pseudo-sterile mice were non-biologically active conjugated forms. In contrast, catecholamines in the intestines of specific pathogen-free mice are biologically active free form (Asano et al., 2012). The levels of catecholamines in the intestines, especially norepinephrine, fluctuate with infection, inflammation, or sympathetic tone. Norepinephrine is an effective bacterial stimulator that can upregulate the proliferation, toxicity, and adhesion of bacteria. However, long-term infection and injury can cause chronic visceral sensitization. Norepinephrine can increase the growth rate, vitality, and invasion of *Campylobacter jejuni*, destroying the tight junctions of the intestinal epithelium (Cogan et al., 2007). Norepinephrine, exudated from the noradrenergic nerve terminal or the capillaries in the intestinal wall, can activate the adrenergic-like QseC receptor on the surface of the bacteria in the intestinal lumen, altering the virulence of the microbiota *via* autoinducer 3-mediated signaling pathways. Simultaneously, the autoinducer three released by gut microbiota into the intestinal lumen can activate intestinal epithelium’s adrenergic receptors, reduce intestinal epithelial cell fluid secretion, and impair the host’s ability to expel pathogens (Rhee et al., 2009). Among the adrenergic receptors, only α2A adrenergic receptors are expressed in ECs, located on the basolateral side, and receiving sympathetic excitatory stimulation. Adrα2A-TRPC4 mediates the catecholamine sensitivity of ECs through a Gαi-dependent signaling cascade (Bellono et al., 2017). The destruction of the gut microbiota induced by antibiotic vancomycin in the neonatal period resulted in visceral allergy in adult male rats, accompanied by decreased mRNA expression of α-2A adrenergic receptor and TRPV1 receptor in the lumbosacral spinal cord (O’Mahony et al., 2014), so α2A adrenergic receptors may act as a chronic pain target for gut microbiota.

## Serotonin Signaling by ECs

Part of the 5-HT secreted by ECs can be used as neurotransmitters for synaptic connection with primary afferent nerve fibers. In contrast, the other part can be circulated to other tissues, including the brain, through blood circulation to participate in osteogenesis, learning, memory, emotional regulation, pain tolerance, etc. It is a crucial signal molecule in the bidirectional communication system between the brain and the gut (Crowell and Wessinger, 2007; Gershon, 2013). A variety of intestinal stimulants, such as mechanical stimulation, diet, gastric acid, bacterial metabolites, viruses, and drugs, can trigger ECs to release 5-HT to intestinal mast cells, spinal afferent nerves, and neurons in the ENS, eventually causing afferent nociceptive and mechanical sensitivity terminal receptor sensitivity, resulting in hyperalgesia (Akiba et al., 2017; Wood, 2020). Under normal physiological conditions, the release of 5-HT can stimulate the intestinal epithelium and ENS, maintain the contraction of intestinal smooth muscle, and facilitate the elimination of harmful bacteria in the intestinal lumen. However, the proliferation of ECs and increased availability of 5-HT are involved in the development of visceral sensitization and peripheral mechanical hyperalgesia in IBS (Cremon et al., 2011; Qin et al., 2019; Nascimento et al., 2021). For example, stimulating FFAR2 on ECs can enhance the defense function of the duodenal mucosa by increasing HCO3^-^ secretion and regulating 5-HT biosynthesis. However, excessive activation of FFAR2, which drives the excessive release of 5-HT, can cause mucosal damage by reducing mucosal blood flow (Akiba et al., 2017). The presence of serotonin transporter knockouts in female mice can also exhibit visceral sensitization and gastrointestinal motility disorders, such as low pain pressure threshold and increased fecal output, accompanied by the increased relative proportion of ECs and colon 5-HT concentration (Bi et al., 2021). The mechanism by which ECs release 5-HT is also related to adenosine triphosphate (ATP) and its metabolites. ATP and its breakdown products are a purinergic transmitter in the ENS, which can initiate enteric nerve reflex or activate afferent nerve endings to transmit pain to the brain. Through BON cells or EC cells isolated from human intestinal surgery specimens, it is verified that ECs can respond to ATP, uridine triphosphate (UTP) and uridine diphosphate (UDP), mainly by activating P2X3, P2Y4R and PLC/IP3/IP3R/SERCA Ca^2+^ signaling pathways, to involve in visceral sensitization and pain production (Xu et al., 2008; Liñán-Rico et al., 2017). In addition, metabolites of norepinephrine and isovalerate activate ECs to release 5-HT (Bellono et al., 2017). And 5-HT can also activate TRPV4 to mediate visceral hypersensitivity through protein kinase C (PKC), phospholipase Cbeta (PLCbeta), mitogen-activated protein kinase kinase (MAPKK) and phospholipase A2 (PLA2)-dependent mechanisms (Cenac et al., 2010).

## Gut Microbiota-ECs-Vagal Afferent Nerves Signaling

**Figure 1:**
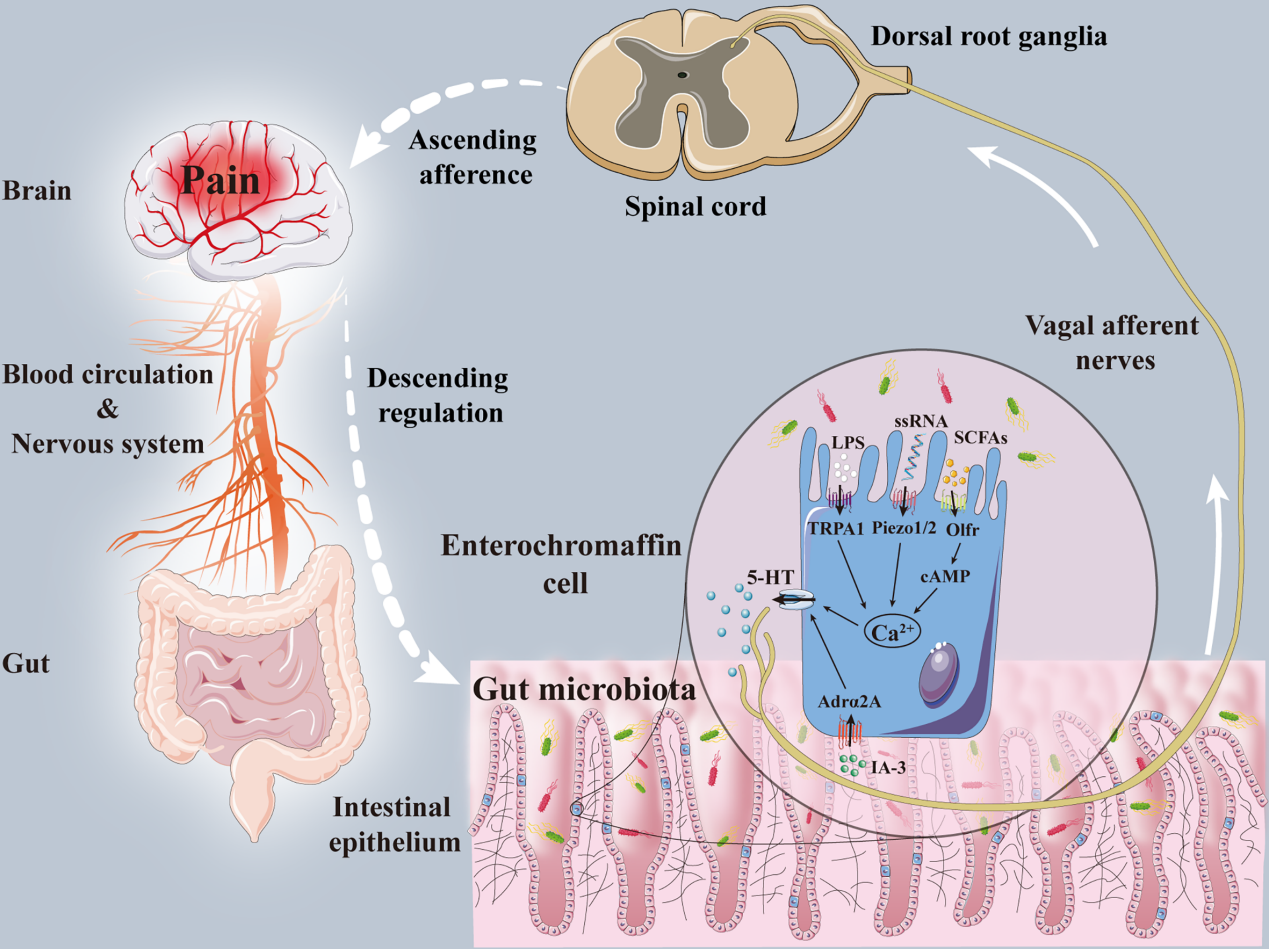
The mechanisms of enterochromaffin cells in the interaction between gut microbiota and pain. Gut microbiota and its metabolites, including IA-3, ssRNA, SCFAs and LPS, acted on enterochromaffin cells receptors (Adrα2A, piezo1/2, cAMP, Olfr, TRPA1) to induce the release of 5-HT. Part of 5-HT transferred hyperalgesia signals to dorsal root ganglia and spinal dorsal horn through the vagal afferent nerve. Pain signals are then transmitted to the brain through the spinal-thalamic tract and other ascending afferent tracts, and eventually integrated through the cerebral cortex and limbic system. After that, the brain can regulate pain sensation through descending regulation and neuromediators. Adrα2A, α2A adrenoreceptor; cAMP, cyclic adenosine monophosphate; IA-3, autoinducer 3; LPS, lipopolysaccharide; Olfr, olfactory receptor; SCFAs, short-chain fatty acids; ssRNA, single-stranded RNA; TRPA1, transient receptor potential A1; 5-HT, 5-hydroxytryptamine.

A large number of studies have suggested that the vagus nerve is one of the key pathways in the mechanism of the gut-brain axis (O’Mahony et al., 2011; Xu et al., 2020). The gastrointestinal tract not only contains a huge microbial ecosystem but also includes ENS composed of tens of thousands of sensory afferent neurons. The enteric vagus nerve originates from the neural crest cells of the vagus nerve and is comprised of nerve plexus embedded in the intestinal wall. Due to its autonomy, neurotransmitter diversity, and complex cell structure, ENS enjoys the “second brain” reputation (Fattahi et al., 2016). ENS is connected with the central autonomic neural network of the brain through the parasympathetic nerves and sympathetic nerves. These sympathetic and parasympathetic nerves can regulate ENS through afferent and efferent activities, thereby forming the bidirectional activity of the gut-brain axis. The intestinal vagus nerve regulates the contraction of gastrointestinal smooth muscle and the secretion of glands, innervates the gastrointestinal mucosal mechanical receptors, chemoreceptors, and tension receptors, and transmits sensations to the nucleus tractus solitarius, and then projects to the central nervous system, such as the amygdala, thalamus, and the locus coeruleus, etc. The colonization of the gut microbiota will also affect the development, excitability, and plasticity of the ENS (Collins et al., 2014; Hyland and Cryan, 2016). The excitability of endogenous primary afferent neurons was reduced, and nitrergic neurons were increased, and calbindin positive neurons and glial cells were decreased in the colon and ileum muscle of GF mice. While the colonization of normal gut microbiota can restore neurons excitability and the number of calbindin positive neurons (McVey Neufeld et al., 2013; Hyland and Cryan, 2016). In addition, colonization of *Bacteroides thetaiotaomicron* restored the growth of neurites and glial cells and the expression of nitric oxide synthase, substance P, and other neurotransmitters (Aktar et al., 2020). The probiotic *Saccharomyces boulardii* reduced the calcium-binding protein intermuscular neurons in pig jejunum (Kamm et al., 2004). *Pediococcus acidilactici* treatment increased galanin and calcitonin gene-related peptide-immunoreactive neurons and glial fibrillary acidic protein-positive enteric glial cells in submucosal ileal ganglion of piglets (di Giancamillo et al., 2010). All the above proved that the gut microbiota can selectively affect the growth and stability of the ENS. Hence, ECs was considered one of those mediators promoting the communication between the gut microbiota and ENS (Bohórquez et al., 2015; Yano et al., 2015). ECs releases 5-HT to regulate the growth, maintenance, and nerve reflex of the intestinal mucosa and ENS by stimulating 5-HT receptors on submucosal primary afferent neurons (Pan and Gershon, 2000; Gross et al., 2012). Clinical data showed that the increased number of ECs was present in the colonic mucosa of IBS patients, and the increased 5-HT released from the mucosa was related to the severity of discomfort such as abdominal pain. Perfusion of colonic mucosal supernatant in IBS patients resulted in significant activation of mesenteric sensory neurons, inhibited by 5-HT_3_ receptor antagonists, indicating that 5-HT released by ECs can affect the ENS in hyperalgesia (Cremon et al., 2011) (**Figure 1**).

Throughout the gastrointestinal tract, there are other enteroendocrine cells except ECs, there are interactions between these cells and ECs, such as hormone stimulation, and so on (Yu et al., 2016). as a limitation, this review discussed only the role of ECs in hyperalgesia, ignoring the effects of other enteroendocrine cells influenced by the ECs after the disturbance of the gut microbiota. Therefore, it is necessary to further investigate the effects of enteroendocrine cells influenced by the ECs. In addition, different gut microbiota may have different effects on ECs, which need to be further studied.

## Conclusion

By sensing different gut microbiota and its metabolites, ECs can activate pain-related receptors and induce the release of 5-HT to transmits pain signals to the brain through the vagus nerve. ECs become sentinels for sensing the gut microbiota to participate in the occurrence of hyperalgesia. Although the characteristics and functions of ECs *in vivo* still need to be further understood, the existing studies have laid a theoretical foundation for the effect of ECs receptors and 5-HT secreted by ECs on the gut microbiota and hyperalgesia. To continue to explore the underlying mechanism of ECs will be an indispensable and promising challenge in the search for pain treatment strategies in the future.

## Author Contributions

XX wrote the manuscript of this review. All authors critically reviewed and approved the final version of the paper.

## Funding

This study was supported by a grant from the National Natural Science Foundation of China (Grant No. 81341034).

## Conflict of Interest

The authors declare that the research was conducted in the absence of any commercial or financial relationships that could be construed as a potential conflict of interest.

## Publisher’s Note

All claims expressed in this article are solely those of the authors and do not necessarily represent those of their affiliated organizations, or those of the publisher, the editors and the reviewers. Any product that may be evaluated in this article, or claim that may be made by its manufacturer, is not guaranteed or endorsed by the publisher.

## Abbreviations

ACC, anterior cingulate cortex; ATP, adenosine triphosphate; BBB, blood-brain barrier; CB, cannabinoid receptor; ECs, enterochromaffin cells; ENS, enteric nervous system; EPSs, exopolysaccharides; FFAR, free fatty acid receptor; GF, germ-free; GPR, G-protein coupled receptor; IBS, irritable bowel syndrome; IL, interleukin; LPS, lipopolysaccharide; Olfr, olfactory receptor; PAMPs, pathogen-associated molecular patterns; SCFAs, short-chain fatty acids; ssRNA, single-stranded RNA; TLR, Toll-like receptors; TRP, transient receptor potentials.
